# Developing a CVTAE-based conceptual framework for examining emotions in higher education teaching: a systematic literature review

**DOI:** 10.3389/fpsyg.2023.1142506

**Published:** 2023-05-05

**Authors:** Nicola A. Maier, Julia Mendzheritskaya, Gerda Hagenauer, Miriam Hansen, Robert Kordts, Melanie Stephan, Katharina Thies

**Affiliations:** ^1^Department of Educational Psychology, Institute of Psychology, Goethe University, Frankfurt, Germany; ^2^School of Education and Institute of Educational Science, School Research and School Practice, Paris Lodron University of Salzburg, Salzburg, Austria; ^3^Department of Education, Faculty of Psychology, University of Bergen, Bergen, Norway; ^4^Institute for Educational Science, Research and Teaching Unit for Pedagogy With a Focus on Media Education, University of Erlangen-Nuremberg, Nuremberg, Germany; ^5^Independent Researcher, Lemgo, Germany

**Keywords:** higher education teachers, systematic review, teaching related emotions, emotion regulation, antecedents and consequences of emotions

## Abstract

A number of studies on higher education (HE) teachers' emotions have been carried out, but overall, the literature on this issue is relatively limited, even though HE teaching can be regarded as an emotional endeavor and represents an important topic in HE research. The main goal of this article was to develop a conceptual framework for examining teaching-related emotions of HE teachers by revising and extending the control-value theory of achievement emotions (CVTAE) developed to systematically classify existing findings on emotions in HE teachers and to identify a research agenda for future studies in this field. Therefore, we conducted a systematic literature review on empirical studies investigating HE teachers' teaching-related emotions to gain insights into (1) the theoretical concepts and approaches used to study HE teachers' emotions as well as the (2) antecedents and (3) consequences of experienced emotions identified in the existing studies. By applying a systematic literature review, 37 studies were found. Based on the conducted systematic review, we propose a CVTAE-based conceptual framework for examining HE teachers' emotions in HE teaching with additional components relating to both antecedents and consequences of HE teachers' experienced emotions. We discuss the proposed conceptual framework from the theoretical perspective, pointing out new aspects that should be considered in future research on HE teachers' emotions. From the methodological perspective, we address aspects related to research designs and mixed-method approaches. Finally, we list implications for future higher education development programs.

## 1. Introduction

Although a growing body of research concerning the emotions of schoolteachers has been conducted over the last 20 years, the emotions of teachers in higher education (HE) have been of little interest to researchers thus far. In 2007, Pekrun stated, “To date, next to nothing is known about professors' emotions experienced in classroom teaching, and the role these emotions play in the quality of their teaching, their professional development, and their wellbeing, burnout, and physical health” (p. 604) (Pekrun, 2007). We argue that HE teachers' emotions should be acknowledged in research because emotions guide behavior and are thus likely to influence the quality of teaching and, as a result, the learning outcomes of HE students. In addition, emotions are linked to personal wellbeing. These associations have been repeatedly confirmed for schoolteachers (Frenzel, 2014), but research on HE teachers is comparatively scarce. We further propose that teaching-related emotions of HE teachers could be particularly salient, as academics must negotiate the demands of multiple roles simultaneously (e.g., Lai et al., 2014), which could be especially emotionally taxing owing to the potential tensions arising between the different roles (e.g., Avargues Navarro et al., 2010). Additionally, HE teachers frequently do not have (much) professional education in the teaching domain and are thus, at best, loosely formally guided in their professionalization processes. It should also be noted that the pressure on HE teachers due to (mandatory) student evaluations is intense and unrelenting (e.g., Roxå and Mårtensson, 2011), in particular when such evaluations co-determine a teacher's academic career. This is likely to cause various negative emotional side effects and tensions, especially for those who have a strong teaching orientation, but are compelled to enhance their research at the same time. In sum, the above-mentioned studies underline the variability of factors linked to teaching-related emotions of HE teachers and make the systematic examination of theoretical approaches and empirical findings touching upon the emotional experiences of HE teachers an important agenda in HE research.

Among existing theoretical models that have been used for examining emotions experienced in achievement and academic settings (e.g., Fredrickson, 2001; Scherer, 2005; Gross, 2010), the most prominent one is the control-value theory of academics emotions (CVTAE, Pekrun, 2006). This theory was developed based on appraisal-orientated approaches to emotions (Moors et al., 2013). It explains (achievement) emotions in educational settings and has been frequently applied in conjunction with the emotions of school students and HE students. This theory states that emotions are evoked based on two antecedent appraisals, namely control and value appraisals. If learners perceive the *environment* as *controllable* and (intrinsically) *valuable*, positive emotions typically occur (e.g., enjoyment). Conversely, if an environment or learning activity is experienced as uncontrollable and relevant, negative emotions usually result (e.g., anxiety or hopelessness). As a consequence, these emotions influence attitudinal changes, motivational, cognitive, and regulatory processes, implying different types of attempts to manage the emerging emotions.

Although CVTAE provides a solid basis for studying emotions in academic contexts and for classifying antecedents and consequences of affective experiences, it focuses predominantly on the affective phenomena of learners. Therefore, a specification and elaboration of a modified conceptual framework including the revision of existing theoretical approaches such as CVTAE seem to be necessary for a systematic examination of various factors connected to the emergence and processing as well as the consequences of emotions experienced by HE teachers. Consequently, we seek to review the existing empirical studies on teaching-related emotions of HE teachers systematically, considering both theoretical approaches and findings in order to extend the CVTAE into a modified conceptual framework that reflects aspects specific to teaching-related emotions within the HE context. Based on the modified conceptual framework, a broader picture can be painted to obtain a systematic and holistic overview of what is already known about HE teachers' emotions and what aspects of teaching-related emotions of HE teachers are still underrepresented in the current studies. Furthermore, methodological issues of research on teaching-related emotions of HE teachers could be analyzed and implications for teaching development programs could be derived.

## 2. Research questions and aim of the study

The main goal of this review was to revise the CVTAE (Pekrun, 2006) to modify the conceptual framework for examining HE teachers' teaching-related emotions by classifying antecedents and consequences specific to HE teachers' emotions. For this purpose, we identified the following research questions for our systematic literature review, including both theoretical issues and empirical findings touching upon HE teachers' teaching-related emotions.

First, we sought to gain insight into the theories and models of emotions used to study the emotions of HE teachers and to check for CVTAE in particular (Pekrun, 2006).

*RQ 1: How widely is CVTAE (Pekrun*, *2006**) used as a theoretical framework for studying HE teachers' teaching-related emotions? What other theoretical concepts and approaches were used in existing studies?*

Second, we were interested in antecedents linked to HE teachers' teaching-related emotional experiences.

*RQ 2: What kinds of antecedents specific to teaching-related emotions experienced by HE teachers were identified in the existing research and how can the revealed antecedents be classified?*

Third, we sought to investigate the consequences of the emotions experienced by HE teachers.

*RQ3: What kinds of consequences were reported as being linked to the experienced teaching-related emotions in HE teachers, and how can the revealed consequences be classified?*

## 3. Methods

In order to answer the research questions outlined above, we conducted a systematic literature search and a stepwise analysis of the selected studies.

### 3.1. Search terms and databases

An initial systematic literature search was undertaken by the main author in May 2020 in selected databases in education and psychology (ERIC, PubPsych, PsycINFO, Web of Science), following the current standards defined in the PRISMA statement (Page et al., 2021). The keywords in their diverse combinations, linked with Boolean Operators (AND/OR), were “emotion,” “affect,” “emotion regulation,” “emotion management,” “HE teaching,” “HE teachers,” “higher education teaching,” “higher education teachers,” “university teaching,” “university teachers,” “university lecturers,” and “university instructors.” Furthermore, following the “standard systematic review practice” (Petticrew and Roberts, 2006, p. 104), experienced researchers in this research field were contacted and asked for further literature suggestions (in this case: the co-authors).

### 3.2. Selection criteria

Formal criteria included that articles (1) were written in English or German and (2) were published between 2000 and 2020. Several researchers around the turn of the millennium emphasized the role of emotions in school teaching as well as in HE teaching (e.g., Hargreaves, 1998, 2000), but also stressed that especially the latter was a largely under-researched area (Martin and Lueckenhausen, 2005). In recent years, there has been a slight increase in publications, which is why we considered it promising to investigate publications within this timespan. Regarding the study design, we picked only studies that (3) were empirical (quantitative, qualitative, or mixed method), as the aim was to provide an evidence-based overview of HE teachers' emotions. Furthermore, we selected only studies (4) whose subjects were HE teachers (and not, e.g., tutors). Regarding the content of the studies, we were solely and explicitly interested in HE teachers' teaching-related emotions. Consequently, we selected studies that (5) examined HE teachers' emotions regarding teaching and excluded studies that addressed constructs other than teaching-related emotions, such as profession-related emotions such as work satisfaction, wellbeing, stress, or burnout (e.g., Azeem and Nazir, 2008; Abdullah and Akhtar, 2016). To ensure the quality of the articles, we included only studies published in (6) peer-reviewed journals due to their trustworthiness in academia and their rigorous review processes (Nicholas et al., 2015).

### 3.3. Study collection process and data extraction

**Figure 1:**
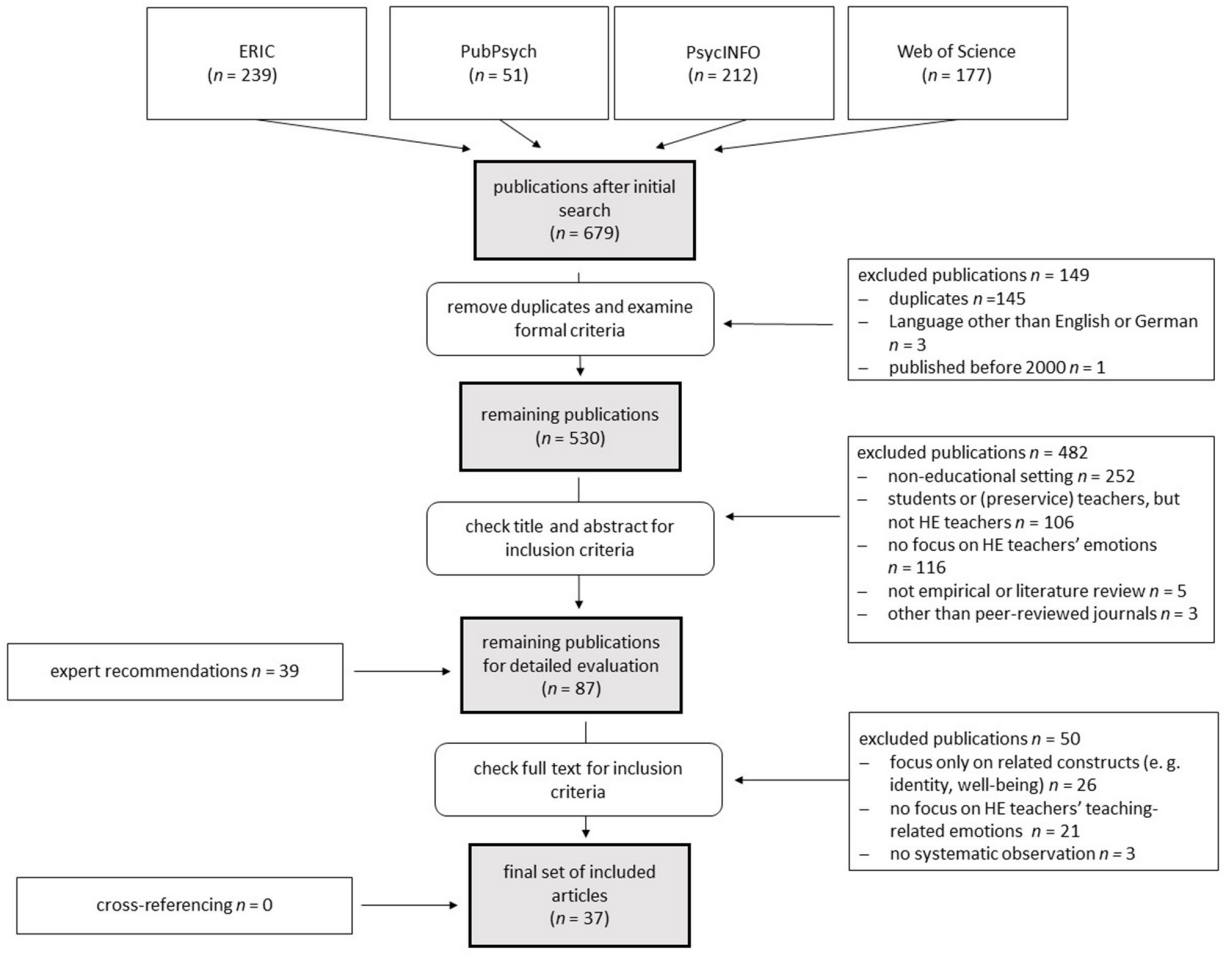
Flowchart of systematic literature search results, number of hits, and reasons for study exclusion.

The study collection process is graphically illustrated in a flow chart (see Figure 1). Altogether, 679 records were identified (239 in ERIC, 51 in PubPsych, 212 in PsycINFO, and 177 in Web of Science [including the SSCI, the SCI, and the A & HCI]). A total of 145 duplicates were removed. The examination for formal criteria lead to the exclusion of four articles (three articles were not written in English or German, and one article was published before 2000). By screening the titles and abstracts of the remaining 530 titles, 482 articles were sorted out gradually: 252 studies did not take place in an educational setting, the subjects of a further 106 articles were not HE teachers (but students or [preservice] teachers, tutors, or other faculty), 116 articles did not focus on HE teachers' emotions, and an additional eight articles were sorted out because they did not appear in peer-reviewed journals (*N* = 3) or were not empirical in nature (*N* = 5). The main author examined full texts of the remaining 48 articles as well as of 39 further articles that experts in this research field recommended for closer examination. If there were doubts regarding the inclusion of articles, at least two authors discussed the respective articles until a consensus was reached. A total of 50 out of these 87 studies were eventually excluded (21 from among the expert recommendations and 29 of the further articles): 26 articles only focused on related constructs (such as identity or wellbeing), 21 articles did not focus on HE teachers' teaching-related emotions, and in three other studies, no systematic observation took place. No further studies could be identified by cross-referencing the included studies. In total, 37 studies were included in this literature review. A summary of the included studies is presented in Table 1, providing information on the research objective, sample and country, empirical approach, and data collection strategy.

**Table 1:** Summary of empirical studies on emotions in HE teaching.

**References**	**Research objective**	**Sample and Country**	**Empirical approach**	**Method/data collection strategy**
Badia et al. (2019)	HE teachers' online teaching related emotions and influencing factors (teaching approach, individual characteristics)	*N* = 965 HE teachers; Spain	Quantitative	Online questionnaire
Badia Gargante et al. (2014)	Structure of HE teachers' emotions about teaching and teaching approaches	*N* = 198 HE teachers; Latin America (Peru/Colombia/Chile/Mexico/Argentina)	Quantitative	Online questionnaire
Bahia et al. (2017)	HE teachers' emotional states as a result of Bologna-related changes	*N* = 12 HE teachers with extensive teaching experience; Portugal	Qualitative	Semi-structured interviews
Bennett (2014)	Emotions of HE teachers applying innovative teaching methods (Web 2.0)	*N* = 16 HE teachers in various disciplines with diverse teaching experience; UK	Qualitative	Interviews
Coppola et al. (2002)	Presents a qualitative study of role changes that occur when faculty become online or “virtual” professors	Instructors in ALN settings; US	Qualitative	Interpretative Study
Cowie (2011)	Antecedents of EFL (English as a Foreign Language) teachers' emotions	*N* = 9 EFL teachers; Japan	Qualitative	Phenomenological interview (3 interviews with each teacher)
Flodén (2017)	HE teachers' perceptions of student feedback and its relation to teaching choices	*N* = 75 HE teachers; Sweden	Quantitative	Online questionnaire
Gates (2000)	HE teachers' handling of emotions during teaching and the relationship to their purpose of facilitating students	*N* = 9 HE professors; US	Qualitative	Observation And interviews
Hagenauer et al. (2016)	Cross-cultural differences in HE teachers' emotion display and teacher-student-relationship	*N* = 15 HE teachers in teacher education; Australia *N*= 9 HE teachers in teacher education; Germany	Qualitative	Semi-structured interviews
Hagenauer and Volet (2014b)	HE teachers' emotion display when teaching and interacting with students	*N* = 15 HE teachers in teacher education; Australia	Qualitative	Semi-structured interviews
Hagenauer and Volet (2014a)	Variety and antecedents of HE teachers' emotions in teaching and interacting with students.	*N* = 15 HE teachers in teacher education; Australia	Qualitative	Semi-structured interviews
Harlow (2003)	Emotions and emotion management of faculty and its variation by race	*N* = 58 (29 white, 29 African-American faculty members at a university with 91 % white student population); US	Qualitative	In-depth interviews
Kordts-Freudinger (2017)	Study 1: HE teachers' emotions and their approaches to teaching Study 2: Emotions of HE teachers, emotion-regulation strategies and approaches to teaching Study 3: HE teachers' emotions and approaches to teaching	Study 1: *N* = 145 HE teachers; Germany Study 2: *N* =198 HE teachers; Germany Study 3: *N* = 76 HE teachers; Australia and New Zealand	Quantitative	Online questionnaire
Kordts-Freudinger and Thies (2018)	HE teachers' emotions, their emotion regulation strategies and approaches to teaching	*N* = 104 HE teachers of different disciplines; Germany	Quantitative	Questionnaire
Kowai-Bell et al. (2012)	(Study 2): Emotional responses of professors to evaluations on the platform “rate my professors”	*N* = 33 pre-tenured and tenured professors; US	Quantitative	Questionnaire / experimental design
Lahtinen (2008)	HE teachers' views on sources, emotions and distress in pedagogical interaction	*N* = 8 HE teachers in various disciplines (7 female); Finland	Qualitative	Interviews
Löfström and Nevgi (2014)	Exploring drawings of HE teachers in order to get an understanding of their emotions in teaching.	*N* =86 academics, participating in a university pedagogy course; Finland	Qualitative	Visual grammar approach (creative approach)
Lutovac et al. (2017)	HE teachers' emotional reactions to student feedback	*N* = 7 pedagogically trained HE teachers with varied experience; Finland	Qualitative	Semi-structured interviews
Martin and Lueckenhausen (2005)	HE teachers' emotions and their conceptual changes in scholarly thinking or practice over a semester	*N* = 31 HE teachers teaching first- or second-year classes in various disciplines; Australia	Qualitative	Interviews
Meanwell and Kleiner (2014)	Emotional dimensions of first-timeteaching	*N* = 86 first-time HE teachers (graduate students); US	Qualitative	Reflection papers
Mendzheritskaya et al. (2018)	Study 1: Cross-cultural comparison of emotional display rules in German and Russian HE teachers	Study 1: *N* = 159 HE teachers; Germany and Russia	Mixed method	Study 1: Experimental design; online questionnaire
	Study 2: Cross-cultural comparison of antecedents of HE teachers' negative and positive affect and its display in teacher-student interactions	Study 2: *N* = 46 HE teachers; Germany and Russia	Quantitative + Qualitative	Study 2: Semi-structured interviews
Myyry et al. (2020)	To examine the emotions higher education teachers associate with assessment	*N* = 16 HE teachers; Finland	Qualitative	Interview study
Nowakowski and Hannover (2015)	Emotional reactions of HE teachers to student course evaluations	*N* = 183 HE teachers; Germany	Quantitative	Experimental designs
Postareff and Lindblom-Ylanne (2011)	Emotions and confidence within six different teacher profiles (teaching approaches)	*N* = 97 HE teachers in various disciplines; Finland	Qualitative	Semi-structured interviews
Quinlan (2019)	Examines emotional episodes in teaching in order to illuminate the underlying moral concerns of higher education teachers	*66 poems, written by N* = 46 HE teachers; UK/US/Canada/Australia	Qualitative	Poems
Ramezanzadeh et al. (2016)	Examine emotions and emotion navigation regarding (in-) authenticity in teaching	*N* = 20 Iranian adjunct teachers; Iran	Qualitative	2 semi-structured interviews
Regan et al. (2012)	Emotions when teaching in an online learning environment (distance learning)	*N* = 6 HE teachers teaching in special-education teacher programs; US	Qualitative	Focus group
Storrie et al. (2012)	Clinical trainers' emotions when dealing with students with mental health issues	*N* = 16 clinical trainers/teachers in the health sciences; Australia	Qualitative	Semi-structured interviews
Stupnisky et al. (2019)	Role of emotions in predicting university faculty teaching and research performance and to validate newly adapted multi-item measures of faculty emotions	*N* =312 Early-career faculty (assistant professors); US	Quantitative	Online questionnaire
Stupnisky et al. (2016)	Variation in new faculty members' emotions and their correlates with perceived teaching and research success	Study 1: *N* = 18; US	Study 1: Qualitative	Focus groups
		Study 2: *N* = 79 pre-tenured faculty members; US	Study 2: Quantitative	Online questionnaires
Thies and Kordts-Freudinger (2019a)	Investigation of relationship between intensity of 4 positive state emotions, different work domains and cognitive appraisals	*N* = 50 university academics; Germany	Quantitative	Online questionnaires
Thies and Kordts-Freudinger (2019b)	Intra-individual analysis of university academics' current state emotions, value and control appraisal dimensions	*N* = 50 university academics; Germany	Quantitative	Online questionnaires
Trigwell (2012)	HE teachers' emotions in teaching and their approaches to teaching	*N* = 175 HE full-time teachers with recent teaching experience; Australia	Quantitative	Online questionnaire
Tunguz (2016)	Emotional labor of academics and its variation due to tenure and gender	*N* = 180 faculty members of 3 Midwestern colleges; US	Quantitative	Online questionnaire
Vannini (2006)	Professors‘(emotional) experience of in-/authenticity in teaching	*N* = 46 (assistant, associate and full) professors; US	Qualitative	Semi-structured in-depth interviews
Wang (2014)	Identify HE teachers‘emotions in order to promote ICT-supported language instruction	Study 1: *N* = 30 EFL HE teachers; Taiwan	Mixed method	Online questionnaire and open ended questions
		Study 2: *N* = 6 EFL HE teachers; Taiwan	Qualitative	Interview
Zhang et al. (2019)	Mediating role of academic self-efficacy in the relationship between emotions in teaching and teaching styles	*N* = 232 academics; China	Quantitative	Online survey

## 4. Results

### 4.1. RQ 1: How widely is CVTAE used as a theoretical framework for studying HE teachers' teaching-related emotions? What other theoretical concepts and approaches were used in existing studies?

To answer RQ 1, we analyzed the theoretical concepts and approaches of emotions used in the included studies (see Table 2). The results showed that CVTAE (Pekrun, 2006) was referred to in 10 out of the 37 included studies.

**Table 2:** Theoretical concepts and approaches of emotions, indicating study authors, years of study, and the number of studies that used this concept/approach.

**Theoretical concept/approach**	**Number of studies, authors and publication year**
Multi-component understanding of emotion (e.g., Kleinginna and Kleinginna, 1981; Scherer, 2005)	6: Trigwell (2012); Badia Gargante et al. (2014); Stupnisky et al. (2016); Kordts-Freudinger (2017); Thies and Kordts-Freudinger (2019a); Myyry et al. (2020)
Dichotomous classifiation of emotions (Kemper, 1978)	1: Zhang et al. (2019)
Appraisal theories (e. g., Moors et al., 2013)	6: Hagenauer and Volet (2014a); Hagenauer et al. (2016); Kordts-Freudinger (2017); Thies and Kordts-Freudinger (2019a,b); Myyry et al. (2020)
Control-value theory of Achievement Emotions (CVTAE; Pekrun, 2006)	10: Hagenauer and Volet (2014b); Löfström and Nevgi (2014); Wang (2014); Hagenauer et al. (2016); Stupnisky et al. (2016, 2019); Quinlan (2019); Thies and Kordts-Freudinger (2019a,b); Myyry et al. (2020) [10]
Broaden-and-build theory of emotions (Fredrickson, 2001)	2: Lutovac et al. (2017); Thies and Kordts-Freudinger (2019a) [2]
Social-psychological approach to emotion (e.g., Zembylas, 2005)	3: Postareff and Lindblom-Ylanne (2011); Hagenauer and Volet (2014a); Zhang et al. (2019)
Emotion regulation strategies (e.g., Gross, 2010)	5: Regan et al. (2012); Hagenauer and Volet (2014b); Hagenauer et al. (2016); Kordts-Freudinger (2017); Kordts-Freudinger and Thies (2018)
Emotional labor/ work (e.g., Hochschild, 1983)	8: Gates (2000); Harlow (2003); Bennett (2014); Hagenauer and Volet (2014b); Meanwell and Kleiner (2014); Hagenauer et al. (2016); Ramezanzadeh et al. (2016); Bahia et al. (2017)
Emotion display rules (e.g., Ekman and Friesen, 1969)	4: Hagenauer and Volet (2014b); Hagenauer et al. (2016); Tunguz (2016); Mendzheritskaya et al. (2018)
No explicit emotion theory/approach used	10: Coppola et al. (2002); Martin and Lueckenhausen (2005); Vannini (2006); Lahtinen (2008); Cowie (2011); Kowai-Bell et al. (2012); Storrie et al. (2012); Nowakowski and Hannover (2015); Flodén (2017); Badia et al. (2019)

Regarding the understanding of what emotions are, the analysis revealed that six out of the 37 included studies that explicitly stressed their multi-componential understanding of emotions. “Multi-componential” refers to the assumption that emotions consist of various emotion components (e.g., Kleinginna and Kleinginna, 1981; Scherer, 2005), which are presumed to interact during an emotional episode. They include cognitive processes (e.g., appraisals and judgments), experiential or affective processes (e.g., positive or negative feelings), physiological processes (e.g., peripheral arousal and central nervous activation), expression (e.g., gestures and facial expressions), and behavioral tendencies (e.g., preparations for action), whereby the affective component is particularly characteristic of emotions (Frenzel et al., 2015). In contrast, one of the included studies referred to a dichotomous understanding of emotions, following Kemper's theory (1978), which divides emotions into positive and negative emotions (Zhang et al., 2019).

Two studies referred to Fredrickson's (2001) broaden-and-build theory of emotion (Lutovac et al., 2017; Thies and Kordts-Freudinger, 2019a). This theory states that experiencing positive emotions broadens one's momentary thought-action repertoires, resulting in the construction of persistent individual resources on different levels (physical, intellectual, social, and psychological). A social-psychological-orientated approach to emotions was used in three studies, insofar that the social nature of teachers' emotions was emphasized (e.g., Postareff and Lindblom-Ylanne, 2011; Hagenauer and Volet, 2014a; Zhang et al., 2019). Teaching can be regarded as a social practice (Zembylas, 2005), and thus teachers' emotions are not only influenced by their own individual reality but also by the social context and the relationships which are formed within it.

Thirteen studies focused on approaches related to different aspects of emotion regulation after experiencing teaching-related emotions, namely emotion regulation strategies (five studies), emotional labor or emotion work (eight studies), and emotion display rules (four studies) [note that two studies made use of all three approaches]. Emotion regulation can be described as an attempt to “influence which emotions we have, when we have them, and how we experience [them]” (Gross, 2010, p. 497). This includes a set of different processes on physiological, behavioral, and cognitive levels (Gross and Thompson, 2007; Gross, 2010), in accordance with the multi-component understanding of emotions described above. These strategies include, e.g., cognitive strategies, such as cognitive reappraisal (i.e., to reinterpret or re-evaluate an emotional situation), or response-focused strategies, such as suppressing the expression of emotions. In a similar vein, emotional labor or emotion work is defined as the effort to display the emotion that is perceived as being the expected emotion (e.g., Hochschild, 1983). This can be achieved through one of the following coping strategies: (1) surface acting (i.e., displaying emotions that are not felt but are perceived as appropriate) or (2) deep acting (i.e., attempting to truly experience the expected emotions). The appropriate *display of emotion* in specific contexts and interaction settings can be defined using Ekman and Friesen's (1969) concept of “emotion display rules.” These cognitively represented rules influence the individual display of emotion in accordance with cultural and/or professional definitions of acceptable display modes for emotions (e.g., Matsumoto et al., 2008; Mendzheritskaya et al., 2018).

Regarding the emergence of emotions, six studies made use of appraisal theories. According to appraisal theories, an emotion is triggered by an individual's evaluation of a corresponding situation. Consequently, the same situation can cause different emotions in different people, depending on their respective appraisals (for an overview, see Moors et al., 2013).

No assignment to any emotion theory could be made in 10 studies. This means that the authors did not point out in an explicit manner what emotion theory or approach is applied as a theoretical framework in their studies.

In sum, 27 out of 37 included studies made use of one or more theoretical approaches when examining HE teachers' emotions. Furthermore, the results demonstrated that 10 studies used CVTAE (Pekrun, 2006) as the theoretical model for investigating the emotions of HE teachers. This renders it the most widely used theory when examining HE teachers' emotions within the included studies.

**Figure 2:**
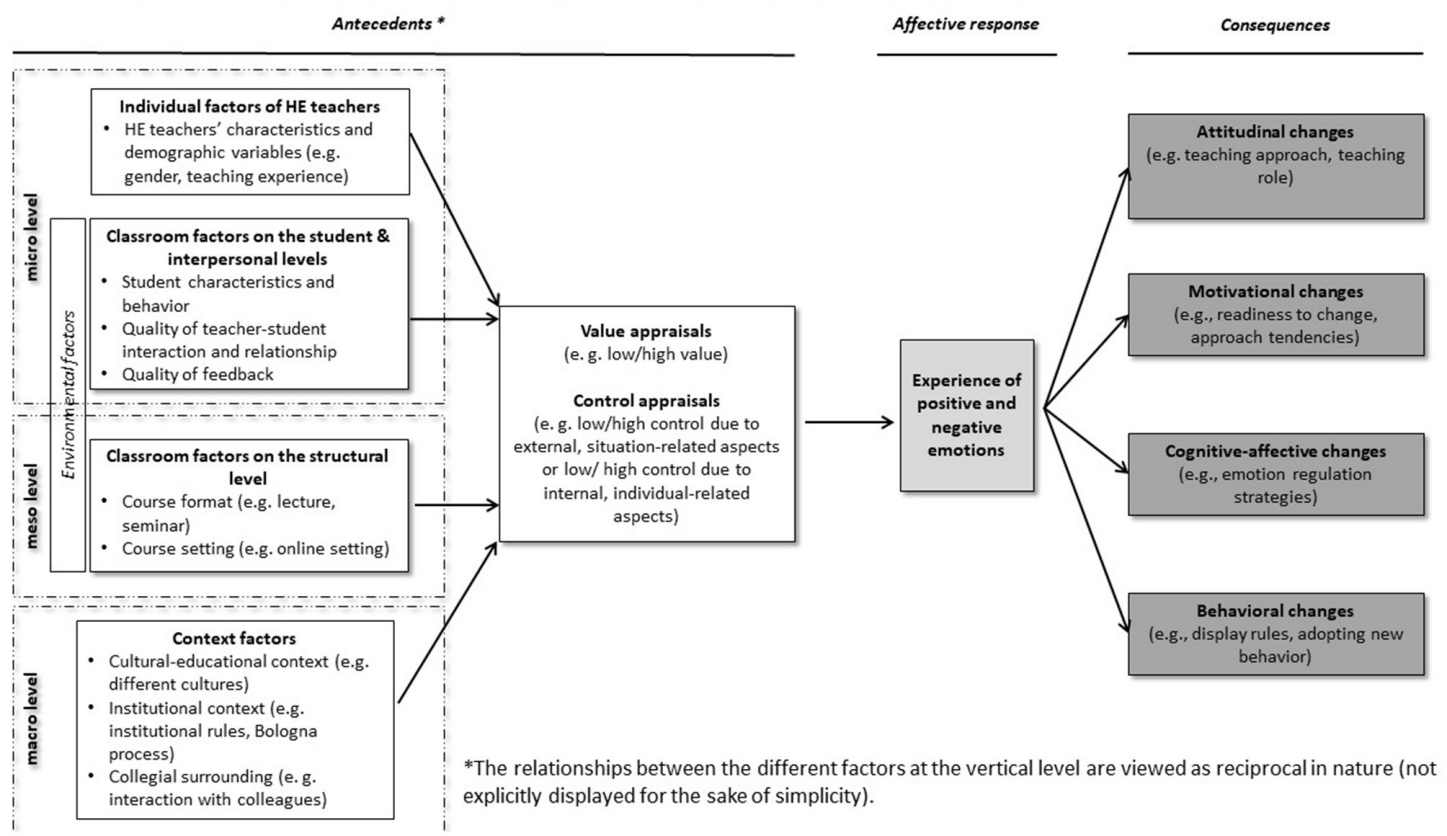
Proposed conceptual framework on studying HE teachers' teaching-related emotions.

Control-value theory of achievement emotions is considered a further development of frequently referenced appraisal theories with an underlying multi-componential understanding of emotions. Additionally, CVTAE allows for a precise subdivision into antecedents and consequences of emotions. On this basis, the results of studies dealing with both antecedents and consequences of HE teachers' emotions can be classified systematically, and the new dimensions specific to emotional experiences in the HE context could be added to the existing components of CVTAE. The undertaken classification of involved studies and identification of new aspects relating to antecedents and consequences of HE teachers' emotions resulted in the proposed conceptual framework (Figure 2). The proposed framework demonstrates how components of the CVTAE can be extended by additional factors connected to antecedents of emotions, affective responses, and consequences of the experienced emotions for a better understanding of the unique characteristics of HE teachers' teaching-related emotions.

### 4.2. RQ 2: What kinds of antecedents specific to teaching-related emotions experienced by HE teachers were identified in the existing research and how can the revealed antecedents be classified?

Following the proposed framework which extends Pekrun's CVTAE (2006), this section is devoted to empirical results on antecedents of emotions. The findings are grouped according to the proposed conceptual framework within the section “antecedents” (cf. Figure 2). From the included studies, we identified different antecedents of HE teachers' emotions on the micro, macro, and meso levels (for an overview, see Fend, 2008) that are related to value and control appraisals of varying intensity. First, we present studies that shed light on individual factors of HE teachers on the micro level (see Section 4.2.1) because the majority of the included studies considered these factors to be relevant variables (or covariates) when analyzing HE teachers' teaching-related experienced emotions, their antecedents, and their consequences. In Section 4.2.2, we analyze results from studies focusing on environmental factors on the micro level (classroom factors on the student and interpersonal levels, see Section 4.2.2.1) and meso level (classroom factors on the structural level, see Section 4.2.2.2). According to CVTAE (Pekrun, 2006), the factors named above influence value and control appraisals and play an important role in the emergence of emotions. We additionally describe broader context factors identified in the studies as antecedents of emotions on the macro level (see Section 4.2.3).

#### 4.2.1. Individual level of HE teachers

Flodén (2017) showed that *teaching experience* plays a role in the perception of feedback, indicating that HE teachers with less teaching experience were more nervous about receiving feedback and reported more negative emotions pertaining to student feedback compared to more experienced teachers. An emotion that often accompanies limited teaching experience is anxiety, or its less intensive forms reflecting fear and insecurity (Hagenauer and Volet, 2014a; Meanwell and Kleiner, 2014), which could be explained by a lower perception of one's own control over the environment. In line with this, performance anxiety can be triggered by other emotions, such as worries about a lack of preparedness (Quinlan, 2019). Related to teaching experiences in general, novel situations of any kind (e.g., teaching in a new position, applying a new teaching method or new technology, teaching a new group of students, and teaching in a new country) can evoke positive emotions (e.g., anticipatory joy), but these typically also go hand-in-hand with feelings of insecurity (Hagenauer and Volet, 2014a). Comparable findings were reported by Lahtinen (2008), who stressed that negative experiences are related to unpredictable and uncertain conditions of teaching and discrepancies in expectations and beliefs that cannot be easily managed. Regarding the use of information and communication technology (ICT), Wang (2014) found that teachers who are less familiar with technology were more nervous and anxious about using ICT in the learning context (e.g., if they were not able to fix the system by themselves). Difficulties encountered with equipment and the resulting loss of time caused frustration. In a similar vein, Bennett (2014) found some HE teachers to be frustrated, infuriated, or desperate if they perceived situations as being out of their control (e.g., if the university's system was not reliable). Furthermore, they experienced fear of exposure if they felt that their own knowledge regarding the technology was not adequate. Regan et al. (2012) confirmed these results by outlining that HE teachers felt disconnected, uncertain, and frustrated because they experienced a lack of control and sufficient technology-based knowledge. Further sources of frustration included HE teachers not being able to adapt their teaching based on students' gestures, questions, or behavior.

Attempting to enhance one's teaching practice can also be regarded as a novel situation, which also can influence the perceived control of HE teachers over their environments. Martin and Lueckenhausen (2005) explored the emotions of Australian HE teachers in one institution who were willing to adopt new teaching practices or rethink their teaching roles. Approaching new practices and roles was accompanied by a mixture of emotions, especially negative ones, such as confusion and anxiety. The emotional impact was particularly observable when the teachers adopted a more student-centered teaching approach. Moving toward a more student-centered teaching approach is a source of insecurity in terms of the more unpredictable nature of student behavior in the classroom.

In an intersectional view, Harlow (2003) showed an interaction between *HE teachers' race* and teaching experience: Less experienced HE teachers with an African American background reported fear, nervousness, and concern regarding “making a mistake” (p. 355). In addition to anxiety, limited teaching experience can also lead to surprising moments that may be either negatively or positively perceived. In general, first-time teachers were surprised by the high emotionality of first-time teaching (Meanwell and Kleiner, 2014). However, “surprise,” but with less negativity, seems to be an emotion also prevalent in the accounts of more experienced teachers (Hagenauer and Volet, 2014a; Kordts-Freudinger, 2017). In general, perceiving oneself as an expert in the field triggers positive emotions, such as enjoyment (Löfström and Nevgi, 2014).

In some studies, a *gender effect* was revealed, e.g., with female teachers reporting more negative emotions associated with feedback from students (Flodén, 2017). Furthermore, male HE teachers reported a slightly higher level of pleasure derived from online teaching as compared to their female counterparts (Badia et al., 2019), and “new” (pre-tenured) female faculty members reported overall higher values for teaching (Stupnisky et al., 2016).

Regarding the *value for teaching compared to research*, there are a few studies that report in part contradictory results: Thies and Kordts-Freudinger (2019b) investigated university instructors' discrete emotions and appraisal antecedents several times a day in a sample of 50 academic staff members in Germany to analyze the state-related emotion-appraisal associations throughout the workday. Their results show that enjoyment, pride, and relief were experienced with a higher intensity in the domain of teaching as compared to the domain of research. More specifically, teaching-related activities such as preparing or holding lectures seem to provoke stronger positive emotions as compared to research-related activities (e.g., the implementation and analyzing of research content). Stupnisky et al. (2019) tested a model of university instructors' discrete emotions for perceived teaching and research success using a single-measurement, retrospective questionnaire. It was shown that the sample of 312 assistant professors on the tenure track in the U.S. and Canada reported overall high levels of enjoyment, moderate levels of anxiety, and low levels of boredom in teaching and research. Furthermore, value appraisals of teaching and research were positively associated with enjoyment and negatively associated with anxiety and boredom. Differing from the results obtained by Thies and Kordts-Freudinger (2019a), HE teachers in this study reported significantly more enjoyment in research than in teaching. However, they experienced less success in research as compared to teaching, which could also explain why faculty reported slightly more anxiety in research. Nevertheless, HE teachers in this study attributed more value to research than to teaching (Stupnisky et al., 2019). Furthermore, the results of a study by Postareff and Lindblom-Ylanne (2011) showed that their sample of Finnish HE teachers typically enjoyed teaching more than marking exams, theses, or doing preparation or post-processing work for their courses (Postareff and Lindblom-Ylanne, 2011). HE teachers in this study disliked lecturing as a particular teaching form most of all, but some teachers also disliked group methods that were activating in nature.

Teachers who express a high identification with the *teaching role* and who are highly committed to teaching typically express very positive emotions with regard to teaching, including passion and “love” for teaching itself, as well as for the subject they are teaching (Postareff and Lindblom-Ylanne, 2011; Bennett, 2014; Hagenauer and Volet, 2014a). In contrast, when HE scholars do not identify with the teaching role but view themselves solely as researchers, they typically experience more emotions that are negative or do not feel emotionally involved at all (Postareff and Lindblom-Ylanne, 2011). More specifically, Vannini (2006) found in his qualitative study on the (emotional) experience of authenticity in teaching that moments of authenticity occurred if teachers valued their teaching roles. However, only minority of the professors in the study stated that teaching was important for their identity. Furthermore, he found that mixed emotions regarding authenticity can occur if professors navigate between their different roles as researcher and teacher. If the professors in the study perceived themselves more as a researcher than a teacher, teaching could feel like a burden, accompanied by frustrated authenticity, boredom, apathy, or even disdain toward students. Regarding the teaching role in online teaching, Badia et al. (2019) associated appropriate emotions in online teaching with teachers' roles. Their results showed that satisfaction and pleasure are associated with teachers who are concerned with ensuring that learners acquire and retain knowledge. In contrast, when the acquisition of content is paramount to the teaching approach, a significant negative relationship between these two emotions was found. Hence, the understanding of roles is important in that teachers who intend to develop students' skills and want to support them find online teaching satisfactory and enjoyable. In contrast, teachers who are instead focused on content and technology aspects are more likely to feel fear and stress.

#### 4.2.2. Environmental factors

The following two sections refer to environmental factors as antecedents of emotions, namely classroom factors. First, study results that we have assigned to classroom factors on the structural level are presented (see Section 4.2.2.1). This is followed by results on classroom factors on the student and interpersonal level (see Section 4.2.2.2).

##### 4.2.2.1. Classroom factors on the structural level

Most important classroom factors considered from a structural perspective cover the aspects of course formats and settings including novel technology-based formats. Regarding the influence of the *course format* on HE teachers' emotions, Löfström and Nevgi (2014) made use of a creative approach in their study. They explored drawings by HE teachers in order to obtain an understanding of their emotions in teaching. Most of the drawings depicted positive emotions (*n* = 40), followed by neutral drawings (*n* = 30), drawings of mixed emotions (*n* = 12), and drawings of negative emotions (*n* = 4). The results showed that emotions in teaching are contextual. While positive emotions (e.g., enjoyment, contemplation, and curiosity) are mostly experienced in small group settings with engaged students and a learner-focused teaching approach, negative emotions (e.g., isolation, anxiety, and discontentment) are more likely to be experienced in lectures with a more content-focused approach due to students' lack of engagement or interest.

The *course setting*, i.e., teaching with new technology, was found to cause mixed emotions, with negative emotions dominating (Regan et al., 2012; Bennett, 2014). In her study on the effect of change processes on HE teachers' emotions, Bennett (2014) found an increased fear of exposure (e.g., not knowing the correct answers to students' online questions), negative emotions due to dependence on technical systems, and fear of humiliation and ridicule (e.g., being laughed at). Potential failure to meet the institution's standards, as evidenced by negative reactions from colleagues, caused an emotional burden on those who applied technology-based teaching methods. Such a change in teaching practice may also require teachers to overcome potentially contrary institutional-cultural norms. The negative emotions found in a study by Regan et al. (2012) on distance learning included that some lecturers felt restricted because everything that they said was recorded, which was perceived as “unnerving” (p. 208); others felt isolated, supporting the importance of social interaction for HE teaching. There were also reported feelings of helplessness or insecurity when lecturers sought to adapt to the role of a “conveyor of information” (p. 210) in the distance-learning setting.

Almost all lecturers in a study on HE teachers' role changes due to the use of an asynchronous web-based learning platform reported that teaching online requires more time and effort, which in turn leads to dissatisfaction or frustration (Coppola et al., 2002). However, the fact that online teaching was experienced as challenging was by no means perceived only in a negative sense. It was precisely this circumstance that stimulated enthusiasm and fascination and challenged creativity (Coppola et al., 2002). Furthermore, teaching with new technology was experienced as convenient and efficient (Coppola et al., 2002; Wang, 2014), e.g., because questions did not have to be answered multiple times (Coppola et al., 2002). Wang (2014) found that HE teachers are generally satisfied when using ICT because they perceive students as more motivated and concentrated as well as being better in interactions and more willing to give answers.

##### 4.2.2.2. Classroom factors on the student and interpersonal level

Students' behavior can have an important impact on HE teachers' emotions. In total, eight of the studies included in this review provide insights into this antecedent of emotions. Disruptive behavior in the classroom and students being late or absent were reported as sources of anger by Japanese HE teachers (Cowie, 2011). In particular, students blaming the teacher in an aggressive way (e.g., for their grading) causes negative feelings in HE teachers and is perceived as a professional identity threat (Lahtinen, 2008). Hagenauer and Volet (2014a) found that limited student engagement (e.g., lack of interest), as well as over-engagement (e.g., being too dominant, not willing to discuss fixed opinions in a constructive manner), can be a source of negative emotions, mainly annoyance. Similarly, Gates (2000) found that HE teachers felt irritated or angry if students behaved in a disruptive manner (e.g., arriving to class late or leaving early without notification, talking to other students instead of listening, and complaining about grades or assignments).

On the other side, students making progress or seeing students succeed was reported as a source of pleasure (Hagenauer and Volet, 2014a), joy (Myyry et al., 2020), satisfaction, and pride (Vannini, 2006). Overall, students and their classroom behavior can challenge a teacher's feeling of passion for teaching. This is reflected in the fact that teachers' joy/passion for teaching varies across different classrooms of students (Hagenauer and Volet, 2014a).

A specific emotional challenge for HE teachers arises from interactions with students who are in personal crisis (Quinlan, 2019) or who exhibit mental health issues, as shown in the study by Storrie et al. (2012) on teachers mentoring a clinical practicum. Dealing with such students can evoke feelings of helplessness and powerlessness, but also fear-related feelings (e.g., due to offensive behavior).

Lahtinen (2008) argues that unrealistic expectations in *teacher–student interaction* can lead to negative feelings. More generally, she notes that the management of relationships between the teacher and students but also among students triggers many emotions in HE teachers (see also Cowie, 2011). In a similar vein, Hagenauer and Volet (2014a) determined that negative emotions arise when expectations with regard to positive teacher–student interactions (e.g., student engagement in class) are not fulfilled (see also Mendzheritskaya et al., 2018) or if students cross boundaries of the teacher–student relationship (e.g., phoning HE teachers during the weekend). Positive emotions of delight or pleasure result if expectations are fulfilled (e.g., motivated students who contribute constructively in class). Harlow (2003) showed that the quality of the teacher–student interaction and ultimately also teachers' emotions differed based on U.S. HE teachers' race, and the interaction between race and gender. HE teachers with an African American background teaching at an American university with a 90% white student population reported greater frustration but also anxiety because they perceived that their intellectual authority (competence and qualification) was more frequently challenged by student behavior (referred to as the “racial double standard”).

Both *receiving feedback from students* and *delivering feedback to students* can be regarded as an emotional issue in the teacher–student interaction. Delivering feedback to students can cause negative feedback for HE teachers if they fear that their pedagogical expertise might be questioned; such an experience can be regarded as a threat to a teacher's identity (e.g., Lahtinen, 2008). Furthermore, grading was experienced less joyful compared to giving formative feedback (Myyry et al., 2020). Boredom was triggered by monotonous assessment methods, whereas joy was experienced if novel summative assessment methods (e.g., learning diaries) were implemented and if students or colleagues supported the assessment practice. Relief was experienced when working together with colleagues, as it enhanced constructive alignment and justice (Myyry et al., 2020).

Higher education teachers reported receiving direct positive feedback from students as a source of positive emotions (Hagenauer and Volet, 2014a). Furthermore, Lutovac et al. (2017) provided evidence of the relevance of student feedback for HE teachers' emotions and their professional development. Positive student feedback was experienced as encouraging, whereas negative feedback was typically perceived as emotionally daunting. Similarly, in an experimental set-up with German HE teachers, Nowakowski and Hannover (2015) determined that qualitative feedback in student evaluations had a stronger influence on emotions than the quantitative data; this was especially true when the students' remarks were negative. Using a vignette approach, Kowai-Bell et al. (2012) found correlations between anonymous ratings on the “Rate My Professors” platform and U.S. professors' (anticipated) emotions, with positive ratings leading to a positive mood and negative ratings, respectively, to a negative mood.

#### 4.2.3. Context factors

As mentioned above, we identified broader context factors on the macro level in the included studies. These factors are related to cognitive appraisals but are also linked to the experiencing of emotions and their consequences (e.g., the expression of emotions). For this reason, the contextual factors are listed as antecedents of emotions but are separated from the environmental factors within the proposed conceptual framework.

Hagenauer et al. (2016) found cultural differences in understanding which HE teachers' emotions are appropriate to express while communicating with students. Especially, the role of cultural-pedagogical context is evident regarding the display of negative emotions (Mendzheritskaya et al., 2018). Furthermore, it was found that the perception of the characteristics of quality teacher–student relationships is likely to vary across cultural-educational contexts, depending on underlying institutional and cultural norms, values, and practices (Hagenauer et al., 2016).

The *institutional context* played a role in the following four studies: Cowie (2011) found that negative emotions were evoked if HE teachers perceived a strong hierarchy or a lack of trust in their institution. In contrast, positive emotions were evoked if the HE teachers had the impression of improvement taking place within the institution. In an interview study by Ramezanzadeh et al. (2016), 20 Iranian adjunct teachers reported on their emotional lives in connection to their perceived authenticity in teaching. In particular, anger was related to attempts to challenge the expectations of the educational system, low salaries, and the inadequate quality of teaching due to existing policies. Quinlan (2019) used poems in order to gain access to teachers' emotions in teaching. She found that emotions can be triggered by institutional rules that should be followed (e.g., no food allowed in the classroom) as well as by self-disclosure (how much of myself should I reveal?). Furthermore, negative emotions might be triggered if the purity of the research subject is violated (e.g., due to the demand of giving “sexy presentations,” p. 1670). Bahia et al. (2017) demonstrated for a sample of Portuguese HE teachers that the Bologna process, i.e., higher education reforms in Europe, triggered ambivalent emotions in HE teachers, but with negative emotions dominating. Many of the interviewed HE teachers described the Bologna process as a threat to their professional identity. Specifically, they experienced their professional autonomy as being threatened, which ultimately caused a decrease in teaching enthusiasm and an increase in sadness, in part because the extended teaching load resulted in less time for individual students. In addition, the HE teachers reported concern and dissatisfaction due to publication pressure, which was experienced as a limitation on their academic freedom. Other emotions that were mentioned were fear and anger. Both dissatisfaction and pressure were experienced as a result of the increasing impact of evaluations, particularly student evaluations.

Higher education teachers interact not only with students but also with their colleagues. This *collegial surrounding* also forms a part of the context. Cowie (2011) study on Japanese HE teachers revealed that interactions with colleagues were often a source of positive emotions, but in case of differences in educational values, could also engender negative emotions. Stupnisky et al. (2016), too, confirmed the relevance of perceived collegiality in the department for HE teachers' emotions in teaching: HE teachers experienced more positive emotions in teaching when collegiality was perceived as better, and when the teachers exhibited personal balance between work and leisure. Moreover, the results of a more recent study by Stupnisky et al. (2019) showed that perceived collegiality predicted value, which in turn negatively predicted teaching anxiety and positively predicted teaching enjoyment.

### 4.3. RQ 3: What kinds of consequences were reported as being linked to the experienced teaching-related emotions in HE teachers, and how can the revealed consequences be classified?

Adopting the well-known proposition of the CVTAE that “achievement emotions affect the cognitive, motivational, and regulatory processes mediating learning and achievement[…]” (Pekrun, 2006, p. 326) for the context of HE teaching, we consider HE teachers' emotional experiences as associated with cognitive, motivational, and regulatory processes linked to teaching practices. CVTAE refers to the so-called consequences of experiencing achievement emotions, stressing the causality and the impact of experienced emotions on individual's cognitive, motivational, and behavioral changes. At the same time, due to the correlative designs applied predominantly in the reviewed studies, it is not possible in each case to infer causal relationships between the analyzed variables from the perspective of the interferential statistics. Therefore, we followed the considerations of the authors regarding the possible effect directions as well as the propositions of the CVTAE concerning the consequences of experienced emotion while summarizing and grouping the research results within our framework into aspects relating to attitudinal changes (Section 4.3.1), motivational changes (Section 4.3.2), changes in cognitive-affective regulation processes (Section 4.3.3), and changes in behavioral regulation processes (Section 4.3.4).

#### 4.3.1. Attitudinal changes

The analysis of the included studies revealed that attitudinal changes as a consequence of HE teachers' teaching-related emotions mainly refer to HE teachers' teaching roles and approaches.

Although research has shown that teaching approaches are significant for teaching behavior and student learning, little is known about the link between HE teachers' emotions and teaching approaches or between HE teachers' emotions and emotion regulation (Kordts-Freudinger, 2017). The few available studies have consistently found a positive correlation between HE teachers' positive emotions or affect and a student-centered approach to teaching, which also includes the establishment of a positive climate in the classroom (Postareff and Lindblom-Ylanne, 2011; Trigwell, 2012; Badia Gargante et al., 2014; Meanwell and Kleiner, 2014; Kordts-Freudinger, 2017; Kordts-Freudinger and Thies, 2018).

The pattern of the interrelationship between HE teachers' negative emotions and their teaching approaches is less clear. Generally speaking, no correlation between negative emotions or affect and a teaching-centered approach has been found (e.g., Trigwell, 2012; Badia Gargante et al., 2014; Kordts-Freudinger, 2017). However, with regard to distinct negative emotions, Kordts-Freudinger (2017) detected a positive correlation between anger and boredom and a teacher-centered teaching approach, whereas Trigwell (2012) revealed a positive association between anxiety and embarrassment and this orientation. Additionally, teachers employing a more teacher-centered approach to teaching exhibited lower levels of pride. The missing overall interrelation between negative emotions and a teacher-centered teaching approach might be traced back to the varying functions of negative emotions in terms of activation or deactivation. Furthermore, the beliefs of teachers regarding professional behavior likely interfere with the direct link between negative emotions and classroom behavior, indicating emotional management. Zhang et al. (2019) found that HE teachers' emotions in teaching, assessed by the *Emotions in Teaching Inventory* (Trigwell, 2012), can directly predict HE teachers' teaching styles. They focused on two so-called “Type-I-teaching styles,” including a legislative and a liberal style, and two “Type-II-teaching styles,” including an executive and a conservative one (see Appendix 1 of the study by Zhang et al., 2019 for key characteristics of the teaching styles). More precisely, the results showed that HE teachers scoring higher on positive emotions tended to use Type I and Type II teaching styles with Type I styles prevailing, whereas HE teachers scoring higher on negative emotions used more Type II teaching styles. They further found that HE teachers' teaching-related emotions indirectly influence their teaching styles through the mediating role of academics' self-efficacy in teaching and research.

The reconsideration of a teacher's role can be a response to their experiencing negative feelings while teaching. Lahtinen (2008) stressed that more intensive reflection on whether the role of the learning facilitator or the role of the learning evaluator is dominant in a teacher's behavior is evoked by unpleasant emotional experiences.

#### 4.3.2. Motivational changes

We found in our analysis that motivational changes as consequences of emotions have only been examined in a few studies, predominantly focusing on the effects of students' feedback or rethinking teachers' roles. The results of the existing research on this matter include the investigation of approach tendencies and readiness to change or improve one's own teaching practice as a consequence of the experienced emotions. Nowakowski and Hannover (2015) showed in a German sample of HE teachers that positive feedback from students' course evaluations was positively connected with an emotional experience of positive valence and negatively linked to the motivation to improve one's future teaching.

Furthermore, if receiving positive student feedback was important to HE teachers, i.e., the value of feedback was high, it influenced the improvement of their teaching (Flodén, 2017). The results of Flodén's study also indicated that HE teachers who reported positive feelings toward receiving student feedback used the feedback more to improve their teaching, as compared to teachers with more negative feelings associated with student feedback. Instead, the latter group rather introduced unnecessary elements in their teaching in order to avoid negative feedback by pleasing students. Next, emotions of anxiety and discomfort experienced while adopting new teaching practices and teaching roles are connected to conceptual change in lecturers' mindsets, including rethinking not only their own but also the students' roles in the teaching-learning process and reflecting on the nature of knowledge and knowledge construction in the subject/discipline (Postareff and Lindblom-Ylanne, 2011).

#### 4.3.3. Cognitive-affective changes

The use of response-focused *emotion regulation strategies*, such as sharing negative emotions with friends or colleagues after negative emotional experiences, was reported by HE teachers in interviews (Hagenauer and Volet, 2014a). Other response-focused regulation strategies reported as reactions to negative emotions experienced were rationalization or acceptance of the situation by the adaptation of expectations (Hagenauer and Volet, 2014a).

Further strategies for dealing with emotions in teaching were observed by Gates (2000), including HE teachers' hiding their emotions or using cognitive strategies such as redefining a situation by holding particular definitions of students or responding selectively to stimuli (e.g., by remembering positive interactions). Another way for HE teachers to prevent the emergence of negative emotions caused by unfavorable student feedback and evaluations is by *distancing themselves* from these emotions (Hagenauer and Volet, 2014a). Distancing oneself from student evaluation seems to become easier as the teaching experience increases, as demonstrated by Kowai-Bell et al. (2012). This phenomenon is reflected in the words of an experienced professor: “After 30 years and tons of reviews, anonymous comments (are) not a big deal” (p. 347).

Some studies highlighted that HE teachers became aware that dealing with emotions associated with student feedback required professional support. Lutovac et al. (2017) showed, for instance, that distancing oneself from students' feedback and reflecting on it more rationally can be learned through pedagogical training and social exchange. The authors note that lecturers are frequently isolated within their departments, amplifying the need for out-of-department pedagogical training opportunities for HE teachers, especially for those at the beginning of their teaching careers (see also Meanwell and Kleiner, 2014).

Few authors considered further factors influencing emotion regulation after experiencing challenging emotions in HE teachers. For example, Bennett (2014) derived from the results of her study that more intensive emotional work is required when difficulties occur external to HE teachers' control as compared to difficulties internal to one's control. Kordts-Freudinger (2017) and Kordts-Freudinger and Thies (2018) found a positive correlation between adaptive emotion regulation (high cognitive reappraisal and low expressive suppression) and a student-centered teaching approach, which is linked to the experience of positive emotions. In comparison, HE teachers with a more teacher-centered approach reported a higher level of emotion suppression.

#### 4.3.4. Behavioral changes

We categorized behavioral changes due to experienced emotions into *display rules* and *adopting new behavior*.

##### 4.3.4.1. Display of emotions

In general, the display of positive emotions as a consequence of experiencing positive emotions occurs often (Hagenauer and Volet, 2014a; Hagenauer et al., 2016; Mendzheritskaya et al., 2018). How teachers communicate their emotions in the classroom can be regarded as a part of their professionalism, as the appropriate communication of emotions fulfills relevant pedagogical functions, including its importance in the shaping of the teacher–student relationships (Hagenauer and Volet, 2014b). Gates (2000) argues that HE teachers also manage their emotions “to model for students particular affective norms” (p. 502) and to use the expression of emotions for socializing students into the preferred role as “questioning, reflective, and responsible learner” (p. 502).

Kordts-Freudinger and Thies (2018) revealed an interrelation between the emotional display and domination teaching approaches. The findings demonstrated a positive link between a student-centered teaching approach and both the controlled display of positive and negative emotions and an uncontrolled display of positive emotions (Kordts-Freudinger and Thies, 2018).

Ramezanzadeh et al. (2016) identified the physical display of emotions as one strategy for dealing with ambivalent emotions. However, other studies found that HE teachers tend to suppress negative emotions due to the belief that the open expression of negative emotions is unprofessional and would interfere with communication and learning (Hagenauer and Volet, 2014a; Hagenauer et al., 2016; Mendzheritskaya et al., 2018). Cultural differences regarding the display rules of HE teachers were examined in a few studies (e.g., Hagenauer and Volet, 2014b; Mendzheritskaya et al., 2018), indicating, e.g., that lecturers in Germany expressed their anger more openly in class compared to their Australian counterparts (Hagenauer and Volet, 2014b), and that Russian lecturers claimed to express their emotions more genuinely than German instructors did (Mendzheritskaya et al., 2018). In addition to cultural differences, there seem to be differences in displaying emotions by the status of faculty members. More specifically, Tunguz (2016) reported that untenured, “low-power” American male faculty reported putting more effort into displaying authoritative emotions (such as anger) when experiencing classroom incivility (e.g., chatting and using mobile phones) in comparison to male faculty who were tenured. Interestingly, this difference was not observed for female faculty. The author traces this finding back to the buffering role of job autonomy in the experience of emotional labor: the traditional gender role (whereby female lecturers are not expected to express authoritative emotions) seemed to impede this effect for female tenured faculty.

##### 4.3.4.2. Adopting new behavior

Coppola et al. (2002) shifted the perspective from the consequences of experiencing emotions to the consequences of expressing emotions. The authors pointed out that the energy and humor that instructors normally experience in the classroom are difficult to convey in asynchronous learning. One consequence of teaching in asynchronous learning networks is that instructors need different tools to express emotions. From the results, it was concluded that online learning environments require better instructional skills, including communication, organization, and motivation (Coppola et al., 2002). Regan et al. (2012), in their study on distance learning, reported that HE teachers experiencing mixed emotions in digital environments use typical problem-oriented coping strategies such as participating in technological training, offering synchronous office hours, or phoning students to deal with their negative emotions.

Ramezanzadeh et al. (2016) found that, in order to deal with ambivalent emotions, faculty members sought dialogue with learners, colleagues, and administrators, and they held internal discourse. In Harlow's study (2003), female professors of color reported investing extensive energy in emotion management, reflecting the merging of two factors: blackness and femaleness. To cope with the fear of making a mistake in front of students, they used coping strategies—among others, overly preparing their lessons (“perfectionism”) and teaching more authoritatively (Harlow, 2003).

## 5. Discussion and conclusion

We conducted the presented systematic literature review to develop a conceptual framework by expanding and revising CVTAE (Pekrun, 2006) for studying teaching-related emotions of HE teachers based on the existing empirical literature. By applying for a systematic literature review, 37 studies were found. First, we analyzed what theoretical concepts and approaches were used in the identified studies for examining HE teachers' teaching-related emotions to gain insight into the approaches used and to explore how widely CVTAE (Pekrun, 2006) was used in this research field (RQ 1). We found that the majority of the included studies (27 out of 37 included studies) made use of one or more theoretical approaches when examining HE teachers' emotions. These results are in line with observations made by Pekrun (2019) that there has been a shift from undertheorized research (see also Quinlan, 2016) toward research that is mostly theory-based. We support Pekrun's (2019) view that this is important for generating a more consistent body of research findings and interpretations. Furthermore, the results demonstrated that 10 out of 37 included studies used CVTAE (Pekrun, 2006) as the theoretical model for investigating the emotions of HE teachers. This represents the highest number of studies applying one specific theory.

Therefore, the components of CVTAE were revised and extended for a new conceptual framework for examining antecedents (RQ 2) and consequences (RQ 3) of HE teachers' teaching-related emotions (see Figure 2). Specifically, additional groups of factors at the micro-, meso-, and macro-levels (for an overview, see Fend, 2008) were integrated into the “antecedents”-component, and differentiated groups of consequences were identified within the “consequences”-component, separated into attitudinal, motivational, affective-cognitive, and behavioral aspects.

Thus, we deduced that environmental factors are antecedents of HE teachers' teaching-related emotions on different levels. We grouped antecedents of emotions into environmental factors on the micro level, i.e., classroom factors on the student and interpersonal level (e.g., students' characteristics and behavior, quality of teacher–student interaction/relationship, quality of feedback to and from students), and on the meso level, i.e., classroom factors on the structural level, including, e.g., the course format (e.g., lecture, seminar) and the setting (e.g., distant teaching / online teaching). Furthermore, the above-mentioned environmental aspects were often analyzed in relation to individual factors of HE teachers on the micro level, such as their demographic variables (e.g., gender, teaching experience). Next, we found that identified factors not only to be related to cognitive appraisals as antecedents of emotions but also to directly influence (to some extent) the experiencing of emotions and their consequences (e.g., the expression of emotions). Additionally, we found broader context factors on the macro level as an antecedent of emotions, i.e., the cultural-educational context (e.g., different cultures), the institutional context (e.g., Bologna process, institutional rules), and the collegial surrounding (e.g., interaction with colleagues). Regarding consequences of experienced emotions in HE teachers, we defined four groups of factors reflecting attitudinal changes relating to teaching roles and approaches, motivational changes linked to readiness to change own teaching practices, cognitive-affective changes associated with emotion regulation, and behavioral changes including the emotional display and adopting new behavior.

Examining the included studies of the systematic review, it can be seen that some areas of the framework have been more addressed in existing studies than others. Much of the reviewed studies cover individual factors and classroom factors on the student and interpersonal levels as well as cognitive-affective changes and behavioral changes in emotions. However, research gaps exist in the area of classroom factors on the structural level, more precisely on course formats. There is also little research on motivational and attitudinal changes due to emotions, which should be addressed more in future studies.

Furthermore, due to the complexity and context specificity of the phenomenon of HE teachers' emotions and emotion regulation, an adequate research methodology is required. Most of the reviewed studies applied a cross-sectional design (generally relying on non-representative samples) and have used either interviews (in various forms) or questionnaires as data collection methods to assess emotions retrospectively. Only Thies and Kordts-Freudinger (2019a,b) assessed in two studies emotions at the moment they occurred and conducted intra-individual analysis. Overall, qualitative studies were more common than quantitative ones (see Table 1). Future research is needed that goes beyond these qualitative and correlational research designs. We would also like to suggest that researchers take advantage of the complementary nature of qualitative and quantitative research on HE teachers' emotions and their regulation by applying mixed-methods designs, including experimental, longitudinal, and within-person research, as suggested as well by Pekrun (2019). This could contribute to gaining a deeper understanding of underlying temporal and causal relations when studying HE teachers' emotions.

In addition, the multi-component nature of emotions permits the use of assessment methods that capture other components, such as physiological, expressive, or motivational aspects of emotion. To the best of our knowledge, no study on HE teaching has yet directly assessed such components. We recommend their inclusion in addition to the methods already employed, to validate past results and to integrate the multi-component perspective upon which current theorizing on emotions is built.

Overcoming the aforementioned methodological issues can provide researchers in the field of HE teachers' emotions with research designs allowing for a more profound examination of causal associations between the different components of the proposed conceptual framework. For instance, the predominantly correlative investigated links between experienced emotions and motivational, cognitive-affective, or behavioral changes in HE teachers could be tested with other research designs including other research and statistical methods.

Future research could also be complemented by social-psychological approaches to emotions (Manstead and Fischer, 2001). They do not only highlight the core role of relationships in emotional interactions (Boiger and Mesquita, 2012), but also suggest an additional appraisal category—namely “social appraisal,” reflecting the fact that “behaviors, thoughts, or feelings of one or more other persons in the emotional situation are appraised in addition to the appraisal of the event *per se*” (Manstead and Fischer, 2001, p. 222).

Furthermore, it would be beneficial to examine the interplay of various antecedent factors identified for the proposed conceptual framework while studying HE teachers' emotions. For instance, when research on HE teachers' emotions and emotion regulation is conducted, the complexity and context specificity of the HE field must be thoroughly considered. Considering the interrelations between cultural factors and pedagogical practices (Volet, 2001) represents a promising approach for investigating the influence of *cultural context* on the affective phenomena of HE teachers. Following this approach, we have argued that what is perceived as an “appropriate” teaching practice in HE (including appropriate emotion display) varies across cultural-educational contexts. Thus, the possibility of generalizing results to other cultural-educational contexts (e.g., disciplines, institutions, and countries) is limited; if attempted, it must be approached with caution and restraint.

The classification of antecedents into micro-, meso-, and macro-levels demonstrated the complementarity of some factors such as classroom factors. Accordingly, we suggest including both the individual perspective focusing on relationships and interaction between teachers and students and the institutional perspective relating to teaching and organizational culture while investigating the role of the classroom factors in the emotional experience of HE teachers. As mentioned above, it should also be kept in mind that academics must negotiate the demands of multiple roles simultaneously (e.g., Lai et al., 2014; Thies and Kordts-Freudinger, 2019a), which might be especially emotionally challenging owing to the potential tensions arising between the different roles (e.g., Avargues Navarro et al., 2010). It should also be noted that due to educational reforms in Europe, i.e., the Bologna process, there is considerable publication pressure and an extended teaching load (Bahia et al., 2017). This is likely to cause various negative emotional side effects and tensions, especially for those who have a strong teaching orientation but are compelled to enhance their personal research qualifications and output as well (e.g., Wilson and Holligan, 2013). This potential conflict provides a battleground for competing emotions. Thus, future research has to take into account the complexity of the institutional field in which HE teachers are placed, with its multiple demands that ultimately could also affect teaching-related emotions (e.g., addressed in the study by Bahia et al., 2017).

As far as the complexity of HE is concerned, not only the Bologna process and other effects of neoliberal policies but also the COVID-19 pandemic have led to sudden changes in the teaching practices of HE teachers. This review has only considered publications until May 2020 and also includes studies that investigated HE teachers' emotions in the context of online teaching/teaching with technology; however, recent research has shown that emergency remote teaching has been experienced as highly emotional by HE teachers (Okoye et al., 2021) and has increased the speed with which digital media and forms of online teaching have been integrated into university teaching in general. This rapid change raises concerns but also hopes in HE teachers (Eringfeld, 2021) and needs further exploration. Considering that only a few studies are addressing the role of teaching format in HE teachers' affective experiences, future studies must take the impact of new technologies and subsequent changes in the HE teaching and learning environment into account.

Based on the results obtained, we would also like to point out some implications for teaching praxis and programs for teaching support. Accordingly, findings connected to different components of the proposed framework (e.g., display rules as a behavioral consequence) should be considered and included when designing professional development programs for HE teachers. This is also important because, e.g., the way HE teachers display their emotions can have an impact on students' learning (Mendzheritskaya and Hansen, 2019). Furthermore, opportunities for social reflection should be created, as sharing and discussing one's teaching-related experiences and emotions can support teachers' development (Pekkarinen et al., 2023). Professional development programs could also contribute to higher levels of perceived control when HE teachers are faced, e.g., with new situations in teaching (especially for HE teachers with little teaching experience), which could in turn lead to the experiencing of more positive teaching-related emotions.

However, this literature review has some limitations. Our database search failed to find articles that experts in this research field recommended for further analysis. Possible explanations could be that we used general search terms such as emotions and affect and did not include specific types of emotions, such as anxiety or physiological reactions such as arousal, and that we only searched four databases. In order to avoid missing relevant articles, the search scope should be expanded in future literature searches. Furthermore, it must be taken into account that we categorized consequences of emotions (e.g., motivational changes due to emotions) based on study results originating from correlational designs that do not allow causal conclusions from the statistical point of view. As noted above, the increased use of mixed-method designs would be fruitful in allowing causal interpretations.

To sum up, CVTAE (Pekrun, 2006) seems to provide a fruitful theoretical foundation to explain not only students' achievement emotions but also teachers' emotions, as teaching in HE can also be regarded as an achievement-related situation. We expanded and revised it for the context of HE teachers. Thereby, we identified additional groups of antecedents that go beyond the environmental factors, i.e., individual factors of HE teachers on the micro level and broader context factors on the macro level, e.g., institutional context factors. Thus, the proposed framework takes specifics of HE teachers (such as the different roles as teachers and researchers) into account that were not addressed in the CVTAE (Pekrun, 2006). We suggest that the proposed CTVAE-based conceptual framework (see Figure 2) could be a productive avenue when examining HE teachers' emotions with new theoretical, methodological, and practical perspectives. With its theoretical and empirical foundation, it could aid in building a starting point for research attempts in this field and could help to close existing research gaps.

## Data availability statement

The original contributions presented in the study are included in the article/supplementary material, further inquiries can be directed to the corresponding author.

## Author contributions

NM reviewed the literature and extracted the data. NM and JM decided on the inclusion of articles and took the lead in writing the manuscript. MH, GH, MS, and KT wrote sections of the manuscript. GH, MH, RK, MS, and KT provided critical feedback and contributed to the discussion. All authors contributed to the conception of the literature review. All authors approved the final version of the manuscript.
